# Reliability and Validity of the Medical Outcomes Study Short Form-12 Version 2 (SF-12v2) in Adults with Non-Cancer Pain

**DOI:** 10.3390/healthcare5020022

**Published:** 2017-04-26

**Authors:** Corey J. Hayes, Naleen Raj Bhandari, Niranjan Kathe, Nalin Payakachat

**Affiliations:** 1 Division of Health Services Research, Psychiatric Research Institute, University of Arkansas for Medical Sciences College of Medicine, Little Rock, AR 72205, USA; cjhayes@uams.edu; 2 Division of Pharmaceutical Evaluation and Policy, Department of Pharmacy Practice, University of Arkansas for Medical Sciences College of Pharmacy, Little Rock, AR 72205, USA; nrbhandari@uams.edu (N.R.B.); nkathe@uams.edu (N.K.)

**Keywords:** non-cancer pain, SF12-v2, reliability, validity, psychometric properties

## Abstract

Limited evidence exists on how non-cancer pain (NCP) affects an individual’s health-related quality of life (HRQoL). This study aimed to validate the Medical Outcomes Study Short Form-12 Version 2 (SF-12v2), a generic measure of HRQoL, in a NCP cohort using the Medical Expenditure Panel Survey Longitudinal Files. The SF Mental Component Summary (MCS12) and SF Physical Component Summary (PCS12) were tested for reliability (internal consistency and test-retest reliability) and validity (construct: convergent and discriminant; criterion: concurrent and predictive). A total of 15,716 patients with NCP were included in the final analysis. The MCS12 and PCS12 demonstrated high internal consistency (Cronbach’s alpha and Mosier’s alpha > 0.8), and moderate and high test-retest reliability, respectively (MCS12 intraclass correlation coefficient (ICC): 0.64; PCS12 ICC: 0.73). Both scales were significantly associated with a number of chronic conditions (*p* < 0.05). The PCS12 was strongly correlated with perceived health (*r* = 0.52) but weakly correlated with perceived mental health (*r* = 0.25). The MCS12 was moderately correlated with perceived mental health (*r* = 0.42) and perceived health (*r* = 0.33). Increasing PCS12 and MCS12 scores were significantly associated with lower odds of reporting future physical and cognitive limitations (PCS12: OR = 0.90 95%CI: 0.89–0.90, MCS12: OR = 0.94 95%CI: 0.93–0.94). In summary, the SF-12v2 is a reliable and valid measure of HRQoL for patients with NCP.

## 1. Introduction

Non-cancer pain (NCP) is a wide-spread debilitating condition with an increasing incidence rate [1,2,3]. The Institute of Medicine (IOM) in 2011 estimated that approximately 100 million Americans were affected by pain, and the cost of its treatment is nearly $600 billion annually [4]. Besides the rising incidence of NCP, the burden to caregivers parallels the disease burden for the patient with NCP. One study found that 70% of patients with NCP reported higher self-perceived burden, achieving a minimally important clinical difference [5]. Caregivers of those with NCP also experience significant burden with impairments in mobility, activities of daily living, and other self-care activities as well as overall level of pain being the largest contributors to higher subjective caregiver burden [6,7].

Since improvements in NCP cannot be measured by laboratory values, clinicians have to rely on subjective assessments of pain to derive effectiveness of any intervention designed to reduce pain. Several pain scales have been validated in the measurement of pain [8,9,10,11]; however, in addition to improvement in pain intensity, patients as well as clinicians hope to see improvements in health-related quality of life (HRQoL). Currently, there is limited long-term evidence on the changes in HRQoL that interventions for NCP may bring. With the current rise in opioid use and abuse [12,13], having validated measures of HRQoL could help ensure that an appropriate benefit to risk ratio is being preserved. One study reported that anxiety, depression, and severe impairment were prevalent within this population [14], and it has been shown that patients with pain of high intensity as well as persistent pain have lower HRQoL [15,16]. Additionally, several studies evaluated the impact of an intervention on HRQoL in those with pain [17,18,19,20].

To date, only four HRQoL instruments have been validated in patients with pain. Vartiainen et al. found the 15D instrument could discriminate pain-related factors and was sensitive in chronic pain patients [21]. Luo et al. validated the Medical Outcomes Study Short Form-12, Version 2 (SF-12v2) in chronic back pain alone, but not in chronic pain overall [22]. The Medical Outcomes Study Short Form-36 (SF-36) and the European Organization for Research and Treatment of Cancer Quality of Life Core Questionnaire (EORTC QLQ-C30) were also validated in patients with chronic nonmalignant pain [23]. Other measures are available to measure pain severity and functionality. Unidimensional scales such as the Visual Analog Scale or the Numeric Rating Scale measure only pain severity. Multidimensional scales such as the Brief Pain Inventory and the Treatment Outcomes of Pain Survey not only measure pain severity but other dimensions affected by pain such as pain interference with life activities and pain coping style [24]. Additionally, performances of the EQ-5D and the SF-6D, which are preference-based measures, have been compared in patients with chronic pain. The EQ-5D appears to have better construct validity and responsiveness in this patient population [25,26].

The SF-12v2 is one of the generic instruments that has been widely used to measure HRQoL in patients with chronic conditions [27,28,29,30,31,32,33]. Since the SF-12v2 is a shorter instrument, it requires less patient and clinician effort [34]. Additionally, the SF-12v2 is embedded in the Medical Expenditure Panel Survey (MEPS), providing an avenue to evaluate HRQoL among patients with NCP on a population level. There is a need to validate the SF12-v2 to help support its use to quantify HRQoL in NCP patients. Therefore, the objective of this study was to evaluate the reliability and validity of the commonly used SF-12v2 in a NCP cohort derived from MEPS.

## 2. Materials and Methods

### 2.1. Data Source

This retrospective cohort study utilized the MEPS data, which is administered by the Agency for Healthcare Research and Quality and is a survey that nationally represents the health of the non-institutionalized, adult US population. MEPS is administered in panels that encompass two years and incorporates five rounds of mail surveys and/or interviews. To compose the panels, households are chosen on a yearly basis from the households that participated in the National Health Interview Survey from the previous year. In order to create national estimates, MEPS provides variables for weighting the data [35].

The Household Component (HC) is one of the prominent constituents of the MEPS data. The HC gathers information from individual household members on general demographics, disease states, overall health status, insurance coverage, charges and payments, employment, income, use and access to healthcare as well as satisfaction with healthcare [35]. The HC also contains the Longitudinal Files. The Longitudinal Files contain the data collected over a two-year time span among participants of a panel. These individuals are interviewed and/or surveyed for five rounds over the two years. For the current study, three panels of the longitudinal files were used including 2010–2011 (panel 15), 2011–2012 (panel 16), and 2012–2013 (panel 17).

### 2.2. Study Sample

We derived a cohort with NCP from MEPS using the definition developed by the IOM in their 2011 report titled Relieving Pain in America [4]. The IOM definition uses four questions in MEPS to identify those with pain: (1) whether or not and to what extend pain interfered with normal work in the past 4 weeks; (2) whether or not the participant, in the past 12 months, has experienced pain, swelling, or stiffness around a joint; (3) whether or not the participant had ever been diagnosed with arthritis; and (4) whether or not the participant had either work or housework limitations. Meeting the criteria for any one of these questions classifies the participant as having pain. Participants defined as having pain from the above definition also had to have the following inclusion criteria: (i) in-scope with data collected from all five rounds of interviews; (ii) eligible for the SAQ (Self-Administered Questionnaire) in both rounds 2 and 4; (iii) no cancer diagnosis in either year of the panel; and (iv) provided responses to the question on pain limitations from the SF-12v2 in both rounds 2 and 4.

### 2.3. Primary Outcome Measure

#### SF-12v2

The SF-12v2 is composed of two component scores: the Mental Component Summary (MCS12) and the Physical Component Summary (PCS12). The MCS12 and the PCS12 measure the latent concepts of mental and physical health, respectively [36]. Each of the components are scored on a scale from 0 to 100 with a mean of 50. Higher scores represent better health. The PCS12 focuses on participants’ general overall health, limitations in mobility, work, and other physical activities as well as limitations due to pain. The MCS12 encompasses participants’ limitations in social activity, emotional state, and level of distraction [37]. Responses to each of the items on the SF-12v2 and its component scores are provided in MEPS. For these analyses, we will use the MCS12 and PCS12 obtained from rounds 2 and 4 of MEPS.

### 2.4. Other Measures

#### 2.4.1. Perceived Health and Perceived Mental Health

In each of the five rounds, two questions are asked that evaluate perceived health and perceived mental health as a part of the Conditions Enumeration section [38]. These two questions are worded as follows: *“In general, compared with other people of the same age, would you say that your (mental health/health) is excellent, very good, good, fair, or poor?”* Perceived health and perceived mental health were used to assess test-retest reliability and construct validity for the PCS12 and MCS12, respectively. The responses to perceived health and perceived mental health were reverse coded to correspond with the direction of the PCS12 and MCS12 [37].

#### 2.4.2. Chronic Conditions

Within round 3 of MEPS, individuals were asked to report their diagnosed conditions. The possible options included emphysema, high blood pressure, diabetes, stroke, asthma, or any heart conditions including angina, coronary heart disease, or heart attack. Diagnoses for heart conditions were aggregated into one binary variable denoting the existence or nonexistence of any heart conditions [32,37,39]. Other chronic conditions were also coded into binary variables denoting the presence or absence of the condition. The number of chronic conditions were summed, which was used for further testing of concurrent validity of the MCS12 and PCS12.

#### 2.4.3. Physical and Cognitive Limitations

With response options being yes or no, individuals, in round 3 of the SAQ, were asked if they had experienced any limitations in the past 3 months in regard to either their physical or cognitive abilities. These two questions were used for evaluating predictive validity of the PCS12 and MCS12 scores from round 2 [37].

#### 2.4.4. Pain Limitation Severity

We determined pain limitation severity using the definition developed by Stockbridge et al. [40], which determined pain limitation severity based on the following question within the SF-12v2 instrument: “*During the past 4 weeks, how much did pain interfere with your normal work (including both work outside the home and housework)?*,” with the response options of “not at all,” “a little bit,” “moderately,” “quite a bit,” and “extremely.” Persons reporting “a little bit” of pain in the first year and at least “a little bit” of pain in the second year were considered as having “a little bit” of pain limitation. Those answering “moderately” in regard to pain level in the first year and at least “a little bit” of pain in the second year were considered to have moderate pain limitations, and those reporting at least “quite a bit” of pain in the first year and at least “a little bit” of pain in the second year were considered to have severe pain limitations. The approach of combining the last two pain response options to derive severe chronic pain was a similar approach to others’ [4]. Pain limitation severity was used in the demographic information and is also a question used in deriving the PCS12 and MCS12 values and their internal consistency.

### 2.5. Statistical Analysis

#### 2.5.1. Reliability

Evaluations of reliability, how well items correlate to each other and contribute to a composite score, conducted in this study were internal consistency and test-retest reliability. Cronbach’s alpha was calculated for internal consistency of the PCS12 and MCS12. Because Cronbach’s alpha does not take into account the multi-dimensionality or weighting of the individual components, Mosier’s alpha was also evaluated [41]. A Cronbach’s or Mosier’s alpha greater than 0.8 represented high internal consistency [41]. Because Mosier’s alpha takes weighting and multidimensionality into account, the value for Mosier’s alpha is hypothesized to be higher than the value obtained with the Cronbach’s alpha. Test-retest reliability of the PCS12 and MCS12 were evaluated using intraclass correlation coefficients (ICCs). An ICC range of 0.4–0.7 was considered moderate and >0.7 was considered to represent high test-retest reliability [42]. To evaluate test-retest reliability, the cohort was restricted to those that reported the same answer to perceived health (N = 7519) and perceived mental health (N = 7500) in rounds 2 and 4. This restriction allows for the evaluation of the test-retest reliability among those that should have similar PCS12 and MCS12 scores between each of the two rounds.

#### 2.5.2. Validity

Construct and criterion validity of the SF12v2 were evaluated. Construct validity, the ability of an instrument to measure the concept it is designed to measure, involved convergent and discriminant validity. Spearman rank correlations were obtained for all correlations between the MCS12, PCS12, perceived mental health, and perceived health for testing of convergent and discriminant validity. Spearman rank correlations were chosen due to perceived mental health and perceived health being ordinal in nature. The magnitude of the Spearman rank correlation coefficient was classified into low (0.1–0.3), moderate (0.3–0.5), and high (0.5–0.7) correlation [43]. For testing convergent validity, we expected to find a moderate to strong correlation between the PCS12 and perceived health since both purport to measure similar aspect of health. Similarly, the MCS12 should moderately or highly correlate with perceived mental health. On the contrary, we hypothesized that the PCS12 and MCS12 would be weakly correlated to each other since they measure a different latent concept, which demonstrated discriminant validity. We also expected to find weak or no correlation between PCS12 and perceived mental health.

Criterion validity evaluated in this study were concurrent and predictive validity. To evaluate concurrent validity, how well a particular measure parallels an established measure of the same construct, a general linear model was used to fit the dependent variable of either PCS12 or MCS12 with the independent variable being number of chronic conditions. The Tukey’s test was also applied to evaluate where the differences lied. Predictive validity, the degree to which the score on one measure predicts the result of another similar measure, was assessed using logistic regression with physical or cognitive limitations from round 3 as the dependent variable with either PCS12 or MCS12 from round 2, respectively.

All missing values were excluded from the final analysis file. A significant *p*-value was set at 0.05. All analyses were conducted within SAS 9.3 (SAS Institute Inc., Cary, NC, USA).

## 3. Results

### 3.1. Participant Characteristics

**Figure 1:**
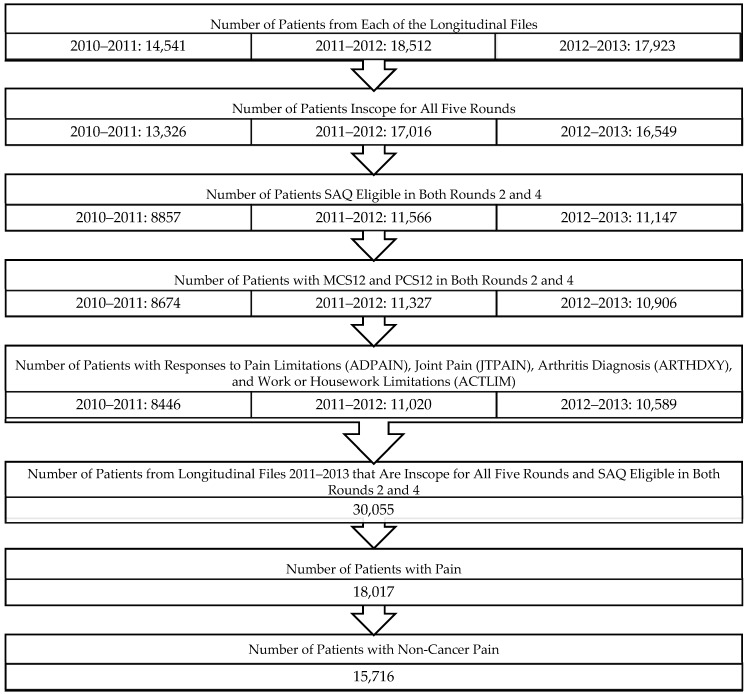
Derivation of study sample.

A total of 18,017 participants with pain were identified from the three panels of the longitudinal files which yielded a final cohort of 15,716 individuals with NCP (Figure 1). Table 1 shows participants’ demographic characteristics in the final NCP cohort. Incorporating weights, the majority were white (80.7%), female (53.4%), and married (52.6%). Most were well-educated with at least some education beyond high-school (55.8%). One-third classified themselves as a middle income group. Most patients had private health insurance (65.4%), but 47.2% did not have prescription medication insurance. Unweighted means for MCS12 and PCS12 from round 2 were 48.9 (SD = 10.9) and 45.8 (SD = 11.3), respectively. Weighted means gave similar scores for both MCS12 (49.6, SE = 0.12) and PCS12 (46.6, SE = 0.14).

### 3.2. Reliability

Cronbach’s alphas for the PCS12 and MCS12 were 0.85 and 0.84 respectively (Table 2). Mosier’s alpha was also evaluated (PCS12 = 0.91, MCS12 = 0.93). Among those with stable perceived mental health, test-retest reliability for MCS12 was moderate (ICC = 0.62, Range: 0.61–0.63), and among those with stable perceived health, test-retest reliability for PCS12 was high (ICC = 0.72, Range: 0.71–0.73) [42]. Therefore, the SF-12v2 is reliable in patients with NCP, with high internal consistency of both PCS12 and MCS12 and high and moderate test-retest reliability of the PCS12 and the MCS12, respectively.

### 3.3. Construct Validity

#### Convergent and Discriminant

The PCS12 was highly correlated with perceived health (r = 0.517) and weakly correlated with perceived mental health (r = 0.241), as hypothesized. The MCS12 was moderately correlated with both perceived mental health and perceived health (r = 0.434 and 0.344, respectively). We found that PCS12 and MCS12 were not correlated with each other (r = 0.029) (Table 3). These findings confirm that PCS12 has high convergent validity with perceived health and high discriminant validity with perceived mental health, establishing strong convergent and discriminant validity for PCS12. Similarly, MCS12 showed moderate convergent validity, since it was moderately correlated with perceived mental health and perceived health. The results also showed that PCS12 and MCS12 measured different concepts of health, as hypothesized. A sensitivity analysis was performed to determine convergent and discriminant validity among those with no other chronic conditions other than NCP. The results of this sensitivity analysis were similar to the results in Table 3.

### 3.4. Criterion Validity

#### 3.4.1. Concurrent Validity

**Figure 2:**
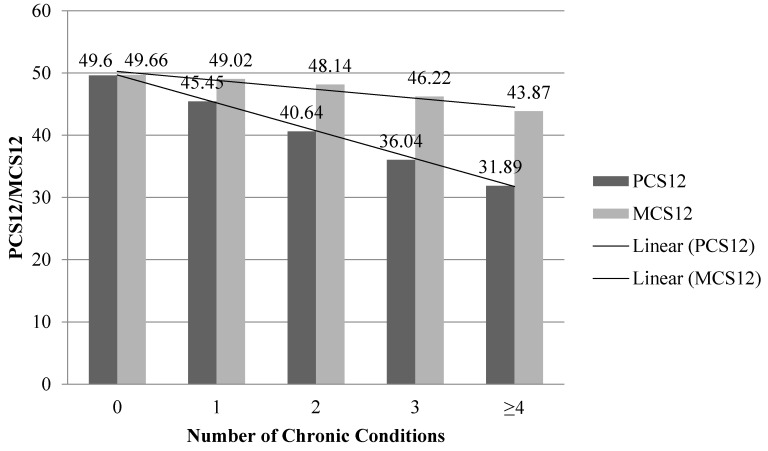
The SF-12 Physical and Mental Component Scores (PCS12 and MCS12) of the study sample, categorized by number of chronic conditions.

Mean PCS12 values ranged from 45.60, among those with no chronic conditions, to 31.89, among those with four or more chronic conditions (Figure 2). Similarly, mean MCS12 values ranged from 49.66, among those with no chronic conditions to 43.87, among those with four or more chronic conditions (Figure 2). Both general linear models were significant (PCS12: *F* = 729.49, *p* < 0.001; MCS12: *F* = 42.91, *p* < 0.001). Using the Tukey’s test, both the PCS12 and MCS12 were significantly lower between each increase in one chronic condition. Therefore, the PCS12 and MCS12 showed strong and moderate concurrent validity with number of chronic conditions, respectively.

#### 3.4.2. Predictive Validity

The PCS12 in round 2 was significantly lower among those reporting physical limitations in round 3, where for each point increase in PCS12 score, there was a 11% lower odds of reporting physical limitations (OR = 0.892, 95%CI: 0.888–0.896). Furthermore, the MCS12 in round 2 was significantly lower among those reporting cognitive limitations in round 3, where for each point increase in MCS12 score, there was a 7% lower odds of reporting cognitive limitations in round 3 (OR = 0.930, 95%CI: 0.925–0.935). Both the PCS12 and MCS12, based on the results of the logistic regressions, showed adequate predictive validity of physical and cognitive limitations, respectively.

## 4. Discussion

Patient-reported outcomes become essential in conditions that do not have objective measures for determining treatment outcomes like NCP. With the prevalence of NCP on the rise [44], as well as the cost of treating it [45], HRQoL of patients with NCP can be used to inform clinicians on treatment selections and also to monitor health outcomes. Information on psychometric properties of HRQoL instruments is crucial to encourage and expand the use of HRQoL instruments in real world settings. This study was the first to evaluate validity of the SF-12v2 for use in patients with NCP using a nationally representative US cohort. We found that the SF-12v2 is valid and reliable for quantifying HRQoL for patients with NCP.

Both the MCS12 and PCS12 demonstrated acceptable internal consistency and test-retest reliability. These findings on internal consistency reliability were similar to a previous study in patients with back pain [22]. To the best of our knowledge, no study evaluating a non-cancer pain population has performed test-retest reliability. Our analysis methods parallel those of Cheak-Zamora et al. who evaluated test-retest reliability of the SF-12v2 in a general cohort of MEPS participants [37]. Our results of test-retest reliability among NCP cohorts were similar to Cheak-Zamora et al. [37].

In evaluating construct validity, the correlations we observed between PCS12/MCS12 and perceived health were similar to Cheak-Zamora et al. [37]. However, our results in a NCP cohort showed the PCS12 and MCS12 to be more discriminant and convergent with perceived mental health, respectively. Vartiainen et al. showed that pain intensity did not predict the score on the 15D instrument [21]. However, we found that both the PCS12 and MCS12 in round 2 were able to predict the likelihood of reporting physical and cognitive limitations in round 3, and therefore, should be able to predict future limitations in physical and mental health.

The findings of MCS12 were similar to those found originally by Ware et al. and Gandek et al. [27]. For concurrent validity, the MCS12, unlike the PCS12, was not significantly lower for each unit increase in the number of chronic conditions but, instead, was only significantly different between those with no and one chronic condition as compared to those with three chronic conditions and those with four or more. The lack of significance between each unit increase in the number of chronic conditions could be due to scale recalibration, where there is either a reprioritization or reconceptualization of quality of life among those with chronic conditions which then effects the MCS12 [46]. Cheak-Zamora et al. also evaluated the concurrent validity of the PCS12 and MCS12 in a non-institutionalized population using number of chronic conditions and found similar behavior [37].

Several limitations exist with this study. First, despite being nationally representative, our sample is predominantly white, well-educated women; therefore, it is unclear whether the SF-12v2 would perform differently in other patient groups with pain. Secondly, these results cannot be generalized to those who are institutionalized with NCP. Thirdly, a comparison of the PCS12 and the MCS12 to the EQ-5D would have been beneficial to this paper; however, the EQ-5D is no longer administered in MEPS. Next, using number of chronic conditions for concurrent validity was limited by the fact that these conditions were not weighted for the differing severity of chronic diseases. Further, the definition for NCP was defined on meeting the criteria on only one of the four MEPS questions. It could be that patients indicating work limitations did not actually have limitations due to NCP, but rather may have limitations due to other causes. The IOM definition for NCP includes work limitations. This criterion may or may not be representative of patients with NCP, as the IOM notes. For this reason, a sensitivity analysis was performed excluding this criteria from the definition. All results for reliability and validity were similar to the main results. Lastly, recall bias and bias due to missing data could be present in survey data like MEPS.

## 5. Conclusions

This study showed that the SF-12v2 was a reliable and valid instrument for measuring HRQoL in patients with NCP. Internal consistency, test-retest reliability, construct (convergent and discriminant) validity, and criterion (concurrent and predictive) validity have been shown to be adequate. In conclusion, the SF-12v2 can be used as a measurement tool to monitor health outcomes in this population. Additionally, HRQoL information obtained from MEPS could be used to inform health status of patients with NCP on a national level.

## Figures and Tables

**Table 1:** Demographic Characteristics of Patients with Non-Cancer Pain.

N = 15,716	Unweighted: N (%)	Weighted: % (SE) ^a^
Age		
18–34	3589 (22.8%)	21.7 (0.52)
35–49	4412 (28.1%)	27.1 (0.53)
50–65	5174 (32.9%)	34.0 (0.63)
66–85	2541 (16.2%)	17.1 (0.54)
Gender		
Male	6875 (43.8%)	46.6 (0.41)
Female	8841 (56.3%)	53.4 (0.41)
Race		
White	10,720 (68.2%)	80.7 (0.85)
Black	3511 (22.3%)	12.3 (0.69)
Other	1485 (9.5%)	7.0 (0.49)
Education Level		
High School or Less	8197 (52.2%)	43.7 (0.72)
More than High School	7384 (47.0%)	55.8 (0.73)
Missing	135 (0.9%)	0.52 (0.07)
Marital Status		
Married	7748 (49.3%)	52.6 (0.77)
Not Married	4044 (25.7%)	24.8 (0.52)
Never Married	3924 (25.0%)	22.6 (0.55)
Health Insurance		
Public	4084 (26.0%)	20.5 (0.64)
Private	8686 (55.3%)	65.4 (0.81)
Uninsured	2946 (18.8%)	14.2 (0.45)
Prescription Insurance		
Insured	6941 (44.2%)	52.8 (0.84)
Uninsured	8775 (55.8%)	47.2 (0.84)
Income Level		
Poor/Near Poor	4243 (27.0%)	20.2 (0.58)
Low Income	2705 (17.2%)	14.4 (0.45)
Middle Income	4556 (29.0%)	30.2 (0.58)
High Income	4212 (26.8%)	35.2 (0.81)
Region		
Northeast	2549 (16.2%)	17.7 (0.71)
Midwest	3213 (20.4%)	22.8 (0.72)
South	5967 (38.0%)	36.9 (0.88)
West	3987 (25.4%)	22.6 (0.69)
Number of Chronic Conditions		
0	7244 (46.1%)	46.8 (0.57)
1	4777 (30.4%)	30.2 (0.46)
2–3	3379 (21.5%)	21.2 (0.42)
≥4	316 (2.0%)	1.8 (0.14)
Pain Limitation Severity		
Mild	2944 (18.7%)	19.8 (0.41)
Moderate	2023 (12.9%)	12.8(0.33)
Severe	2590 (16.5%)	14.5 (0.38)
	Mean (SD) ^d^	Weighted Mean (SEM) ^e^
MCS ^b^ in Round 2	48.9 (10.9)	49.6 (0.12)
PCS ^c^ in Round 2	45.8 (11.3)	46.6 (0.14)

^a^ SE = Standard Error, ^b^ MCS = Mental Component Score, ^c^ PCS = Physical Component Score, ^d^ SD = Standard Deviation, ^e^ SEM = Standard Error of the Mean.

**Table 2:** Internal Consistency and Test-Retest Reliability of the Medical Outcomes Study Short Form-12 Version 2 (SF-12v2).

Reliability	PCS12	MCS12
Standardized Cronbach’s Alpha ^a^	0.85	0.84
Mosier’s Alpha	0.91	0.93
ICC ^b^	0.72 (0.71–0.73) ^c^	0.62 (0.61–0.63) ^c^

^a^ Evaluated from Round 2 of the Medical Expenditure Panel Survey (MEPS), ^b^ ICC = Intraclass Correlation Coefficient, ^c^ Lower and Upper Bounds of ICC.

**Table 3:** Construct Validity (Convergent and Discriminant) of PCS-12 and MCS-12 in SF-12v2 ^a^.

Domain	PCS12 in Round 2	MCS12 in Round 2	Perceived Health	Perceived Mental Health
**PCS12 in Round 2**	1.000	0.029	0.517	0.241
**MCS12 in Round 2**	0.029	1.000	0.344	0.434
**Perceived Health**	0.517	0.344	1.000	0.546
**Perceived Mental Health**	0.241	0.434	0.546	1.000

^a^ Spearman correlation coefficients were classified into low (0.1–0.3), moderate (0.3–0.5), and high (0.5–0.7).

## References

[1] Freburger J.K., Holmes G.M., Agans R.P., Jackman A.M., Darter J.D., Wallace A.S., Castel L.D., Kalsbeek W.D., Carey T.S. (2009). The rising prevalence of chronic low back pain. Arch. Intern. Med..

[2] Zondervan K.T., Yudkin P.L., Vessey M.P., Dawes M.G., Barlow D.H., Kennedy S.H. (1999). Prevalence and incidence of chronic pelvic pain in primary care: Evidence from a national general practice database. Br. J. Obstet. Gynaecol..

[3] Gran J.T. (2003). The epidemiology of chronic generalized musculoskeletal pain. Best Pract. Res. Clin. Rheumatol..

[4] (US) W (DC): NAP Relieving Pain in America: A Blueprint for Transforming Prevention, Care, Education, and Research-PubMed-NCBI 2011. http://www.ncbi.nlm.nih.gov/pubmed/22553896.

[5] Kowal J., Wilson K.G., McWilliams L.A., Péloquin K., Duong D. (2012). Self-perceived burden in chronic pain: Relevance, prevalence, and predictors. Pain.

[6] Jones S.L., Hadjistavropoulos H.D., Janzen J.A., Hadjistavropoulos T. (2011). The relation of pain and caregiver burden in informal older adult caregivers. Pain Med..

[7] Jacobi C.E., van den Berg B., Boshuizen H.C., Rupp I., Dinant H.J., van den Bos G.A.M. (2003). Dimension-specific burden of caregiving among partners of rheumatoid arthritis patients. Rheumatology.

[8] Ferreira-Valente M.A., Pais-Ribeiro J.L., Jensen M.P. (2011). Validity of four pain intensity rating scales. Pain.

[9] Jensen M.P., Turner J.A., Romano J.M.F.L. (1999). Comparative reliability and validity of chronic pain intensity measures. Pain.

[10] Jensen M.P., Miller L., Fisher L.D. (1998). Assessment of pain during medical procedures: A comparison of three scales. Clin. J. Pain.

[11] Bryce T.N., Budh C.N., Cardenas D.D., Dijkers M., Felix E.R., Finnerup N.B., Kennedy P., Lundeberg T., Richards J.S., Rintala D.H. (2007). Pain after spinal cord injury: An evidence-based review for clinical practice and research. Report of the National Institute on Disability and Rehabilitation Research Spinal Cord Injury Measures meeting. J. Spinal Cord Med..

[12] Brady K.T., McCauley J.L., Back S.E. (2016). Prescription Opioid Misuse, Abuse, and Treatment in the United States: An Update. Am. J. Psychiatry.

[13] Frenk S.M., Porter K.S., Paulozzi L.J. (2015). Prescription opioid analgesic use among adults: United States, 1999–2012. NCHS Data Briefs.

[14] Lee S., Chen P.P., Lee A., Ma M., Fong C.M., Gin T. (2005). A prospective evaluation of health-related quality of life in Hong Kong Chinese patients with chronic non-cancer pain. Hong Kong Med. J..

[15] Jonsdottir T., Aspelund T., Jonsdottir H.G.S. (2014). The relationship between chronic pain pattern, interference with life and health-related quality of life in a nationwide community sample. Pain Manag. Nurs..

[16] Dysvik E., Lindstrøm T.C., Eikeland O.J.N.G. (2004). Health-related quality of life and pain beliefs among people suffering from chronic pain. Pain Manag. Nurs..

[17] Björnsdóttir S.V., Arnljótsdóttir M., Tómasson G., Triebel J., Valdimarsdóttir U.A. (2016). Health-related quality of life improvements among women with chronic pain: Comparison of two multidisciplinary interventions. Disabil. Rehabil..

[18] Karmakar M.K., Samy W., Li J.W., Lee A., Chan W.C., Chen P.P., Ho A.M. (2014). Thoracic paravertebral block and its effects on chronic pain and health-related quality of life after modified radical mastectomy. Reg. Anesth. Pain Med..

[19] Joos B., Uebelhart D., Michel B.A., Sprott H. (2004). Influence of an outpatient multidisciplinary pain management program on the health-related quality of life and the physical fitness of chronic pain patients. J. Negat. Results Biomed..

[20] Dysvik E., Kvaløy J.T., Stokkeland R., Natvig G.K. (2010). The effectiveness of a multidisciplinary pain management programme managing chronic pain on pain perceptions, health-related quality of life and stages of change—A non-randomized controlled study. Int. J. Nurs. Stud..

[21] Vartiainen P., Heiskanen T., Sintonen H., Roine R.P., Kalso E. (2016). Health-related quality of life and burden of disease in chronic pain measured with the 15D instrument. Pain.

[22] Luo X., George M.L., Kakouras I., Edwards C.L., Pietrobon R., Richardson W., Hey L. (2003). Reliability, validity, and responsiveness of the short form 12-item survey (SF-12) in patients with back pain. Spine.

[23] Fredheim O., Borchgrevink P., Saltnes T., Kaasa S. (2007). Validation and comparison of the health-related quality-of-life instruments EORTC QLQ-C30 and SF-36 in assessment of patients with chronic nonmalignant pain. J. Pain Symptom Manag..

[24] Younger J., McCue R., Mackey S. (2009). Pain outcomes: A brief review of instruments and techniques. Curr. Pain Headache Rep..

[25] Torrance N., Lawson K.D., Afolabi E., Bennett M.I., Serpell M.G., Dunn K.M., Smith B.H. (2014). Estimating the burden of disease in chronic pain with and without neuropathic characteristics: Does the choice between the EQ-5D and SF-6D matter?. Pain.

[26] Obradovic M., Lal A., Liedgens H. (2013). Validity and responsiveness of EuroQol-5 dimension (EQ-5D) versus Short Form-6 dimension (SF-6D) questionnaire in chronic pain. Health Qual. Life Outcomes.

[27] Gandek B., Ware J.E., Aaronson N.K., Apolone G., Bjorner J.B., Brazier J.E., Bullinger M., Kaasa S., Leplege A., Prieto L. (1998). Cross-validation of item selection and scoring for the SF-12 Health Survey in nine countries: Results from the IQOLA Project. International Quality of Life Assessment. J. Clin. Epidemiol..

[28] Jenkinson C., Layte R., Jenkinson D., Lawrence K., Petersen S., Paice C., Stradling J. (1997). A shorter form health survey: Can the SF-12 replicate results from the SF-36 in longitudinal studies?. J. Public Health Med..

[29] Lim L.L., Fisher J.D. (1999). Use of the 12-item short-form (SF-12) Health Survey in an Australian heart and stroke population. Qual. Life Res..

[30] Lundberg L., Johannesson M., Isacson D.G., Borgquist L. (1999). The relationship between health-state utilities and the SF-12 in a general population. Med. Decis. Making.

[31] Sugar C.A., Sturm R., Lee T.T., Sherbourne C.D., Olshen R.A., Wells K.B., Lenert L.A. (1998). Empirically defined health states for depression from the SF-12. Health Serv. Res..

[32] Ware J., Kosinski M., Keller S.D. (1996). A 12-Item Short-Form Health Survey: Construction of scales and preliminary tests of reliability and validity. Med. Care.

[33] Lacson E., Xu J., Lin S.-F., Dean S.G., Lazarus J.M., Hakim R.M. (2010). A comparison of SF-36 and SF-12 composite scores and subsequent hospitalization and mortality risks in long-term dialysis patients. Clin. J. Am. Soc. Nephrol..

[34] Failde I., Medina P., Ramírez C., Arana R. (2009). Assessing health-related quality of life among coronary patients: SF-36 vs. SF-12. Public Health.

[35] Agency for Healthcare Research and Quality Medical Expenditure Panel Survey Home. http://meps.ahrq.gov/mepsweb/.

[36] Ware J., Lincoln R.I. (2005). How to Score Version 2 of the SF-12 Health Survey (with a Supplement Documenting Version 1).

[37] Cheak-Zamora N.C., Wyrwich K.W., McBride T.D. (2009). Reliability and validity of the SF-12v2 in the medical expenditure panel survey. Qual. Life Res..

[38] Agency for Healthcare Research and Quality Medical Expenditure Panel Survey Questionnaire Sections. https://meps.ahrq.gov/survey_comp/survey_results_ques_sections.jsp?Section=CE&Year1=AllYear&Submit1=Search.

[39] Natarajan S., Nietert P.J. (2004). Hypertension, diabetes, hypercholesterolemia, and their combinations increased health care utilization and decreased health status. J. Clin. Epidemiol..

[40] Stockbridge E.L., Suzuki S., Pagán J.A. (2015). Chronic pain and health care spending: An analysis of longitudinal data from the Medical Expenditure Panel Survey. Health Serv. Res..

[41] Mosier C.I. (1943). On the reliability of a weighted composite. Psychometrika.

[42] Chung L., Valenzuela A., Fiorentino D., Stevens K., Li S., Harris J., Hutchinson C., Assassi S., Beretta L., Lakshminarayanan S. (2015). Validation of a novel radiographic scoring system for calcinosis affecting the hands of patients with systemic sclerosis. Arthritis Care Res..

[43] Hoefman R.J., van Exel N.J.A., Looren de Jong S., Redekop W.K., Brouwer W.B. (2011). A new test of the construct validity of the CarerQol instrument: Measuring the impact of informal care giving. Qual. Life Res..

[44] Nelson L.S., Perrone J. (2012). Curbing the opioid epidemic in the United States: The risk evaluation and mitigation strategy (REMS). JAMA.

[45] Smith M., Davis M.A., Stano M., Whedon J.M. (2013). Aging baby boomers and the rising cost of chronic back pain: Secular trend analysis of longitudinal Medical Expenditures Panel Survey data for years 2000 to 2007. J. Manip. Physiol. Ther..

[46] Blome C., Augustin M. (2015). Measuring change in quality of life: Bias in prospective and retrospective evaluation. Value Health.

